# Comparing the prevalence of statistical reporting inconsistencies in COVID-19 preprints and matched controls: a registered report

**DOI:** 10.1098/rsos.202326

**Published:** 2023-08-16

**Authors:** Robbie C. M. van Aert, Michèle B. Nuijten, Anton Olsson-Collentine, Andrea H. Stoevenbelt, Olmo R. van den Akker, Richard A. Klein, Jelte M. Wicherts

**Affiliations:** Department of Methodology and Statistics, Tilburg University, Tilburg, The Netherlands

**Keywords:** statistical inconsistencies, COVID-19, preprint, registered report

## Abstract

The COVID-19 outbreak has led to an exponential increase of publications and preprints about the virus, its causes, consequences, and possible cures. COVID-19 research has been conducted under high time pressure and has been subject to financial and societal interests. Doing research under such pressure may influence the scrutiny with which researchers perform and write up their studies. Either researchers become more diligent, because of the high-stakes nature of the research, or the time pressure may lead to cutting corners and lower quality output. In this study, we conducted a natural experiment to compare the prevalence of incorrectly reported statistics in a stratified random sample of COVID-19 preprints and a matched sample of non-COVID-19 preprints. Our results show that the overall prevalence of incorrectly reported statistics is 9–10%, but frequentist as well as Bayesian hypothesis tests show no difference in the number of statistical inconsistencies between COVID-19 and non-COVID-19 preprints. In conclusion, the literature suggests that COVID-19 research may on average have more methodological problems than non-COVID-19 research, but our results show that there is no difference in the statistical reporting quality.

## 1. Introduction

The COVID-19 pandemic has led to an exponential increase of publications and preprints (i.e. scientific manuscripts published in open registries that have yet to undergo peer review) concerning COVID-19 [1,2]. Not only is the volume of output almost unprecedented, COVID-19-related articles are also published much faster than their non-COVID-19 counterparts [3]. It is encouraging to see the speed with which the scientific community has responded to this pandemic, but this ‘high-speed’ science may not be without risks. A substantial number of scientists have voiced their concern that the pressure to disseminate findings quickly may decrease scrutiny in performing, reporting and reviewing COVID-19 studies [3–8].

Several empirical studies underline the possible risks of publishing science under pressure. For example, past bibliometric analyses showed low international collaboration in research on natural disasters (including the severe acute respiratory syndrome coronavirus SARS-CoV outbreak in 2003; [9–11]). Furthermore, analyses of the current COVID-19 literature showed poor methodology and reporting in both preprints and peer-reviewed publications (e.g. small samples, lack of control groups, no mention of a sampling frame or study limitations; [12–15]). In a more direct comparison, a recent preprint reported lower methodological rigour in 539 published COVID-19 papers compared with papers published in the previous year in the same journal as the control group [16]. Specifically, Jung *et al*. [16] measured methodological rigour using standard quality checklists such as the Cochrane risk of bias tool [17] and the Newcastle–Ottawa scale [18]. They showed that only 41% of the COVID-19 articles could be considered of high methodological quality, as compared with over 73% of the articles in the control group.

To our knowledge, one important aspect of research quality of COVID-19 studies has not been looked at yet: the quality of statistical reporting. Statistics underlie many conclusions presented in COVID-19 studies, so it is of paramount importance that these statistics are correctly reported. Inconsistent statistics affect the robustness of a conclusion: if a reported statistic is not in line with other information in the paper, the trust in the claim will be lowered. Imagine, for example, that researchers study the impact of a treatment on the mortality of patients infected with the virus. The researchers report an odds ratio, but the odds ratio is not in line with the number of patients in the experimental and control group that die. Such an inconsistency will lower the trust in the finding and corresponding substantive conclusions and policy.

Previous research has shown a high prevalence of statistical reporting inconsistencies in publications from different fields [19–23]. Based on the observed suboptimal methodological and reporting quality of COVID-19 studies, and given the increased time pressure and strong financial and societal interests under which this research is conducted, it is imaginable that the prevalence of statistical reporting inconsistencies in COVID-19 papers is even higher than in non-COVID-19 papers. However, the contrary could also be true: the severity of the pandemic, the perceived importance of COVID-19 research and the expected attention to this research may cause researchers to take more care in reporting the statistical results than they would normally do. To study this, we compared the prevalence of statistical reporting inconsistencies in COVID-19 preprints and matched controls. Specifically, we tested the following hypothesis:

### 1.1. Hypothesis: the prevalence of statistical reporting inconsistencies differs between COVID-19 and matched non-COVID-19 preprints

We specifically focused on preprints for two main reasons. First, preprints have played a central role in early dissemination of scientific insights in the COVID-19 pandemic [2]. Second, preprints are easy to access because they are not behind paywalls and the majority of preprints are published at dedicated preprint servers, which means that they can easily be located and accessed.

## 2. Methods

We studied the prevalence of statistical reporting inconsistencies in COVID-19 as compared with non-COVID-19 preprints. This makes our study a natural experiment, because we are comparing two existing groups that occurred naturally and cannot be controlled. A summary of the design can be found in table 1. All data and code for this project are available on the Open Science Framework (OSF) on https://osf.io/tdfgq/. This paper is a Registered Report. The accepted Stage 1 manuscript is available at https://doi.org/10.17605/OSF.IO/WCND4. The date of Stage 1 in principle acceptance was 24 February 2021. This study was approved by the Ethics Review Board of the Tilburg School of Social and Behavioural Sciences at Tilburg University (reference: RP330).

**Table 1.** Design.

question	hypothesis	sampling plan (e.g. power analysis)	analysis plan	interpretation given different outcomes
Does the prevalence of statistical reporting inconsistencies differ between COVID-19 and non-COVID-19 preprints?	The prevalence of statistical reporting inconsistencies differs between COVID-19 and non-COVID-19 preprints.	Our resources allow us to study 1200 COVID-19 preprints and 1200 non-COVID-19 preprints. This gives us 80% power to detect an effect if the odds ratio of a statistical inconsistency in a COVID-19 preprint versus a non-COVID-19 preprint is about 1.38. See the power analysis section in the electronic supplementary material for more details.	We will analyse the data using two logistic multi-level models. The first model only contains the predictor of interest (COVID-19 or not) and the second model also includes relevant control variables. The hypothesis will be tested in both models using a two-tailed test with *α* = 0.05 and a Bayes factor. See the Analysis section for more details.	We will report the odds ratio (and corresponding confidence interval) that describes how much larger (or smaller) the odds are for reporting a statistical inconsistency in a COVID-19 preprint compared with a non-COVID-19 preprint.
				In the case that the null-hypothesis cannot be rejected in the frequentist hypothesis test, we will conclude that there is no evidence for a difference in the prevalence of statistical reporting inconsistencies between COVID-19 and non-COVID-19 preprints.
				The Bayes factor will be interpreted with a statement along the lines of: ‘the model where the prevalence in statistical reporting inconsistencies differs for COVID-19 and non-COVID-19 preprints is X times more likely than the model where the prevalence is the same for both COVID-19 and non-COVID-19 preprints'.

### 2.1. Population of preprints under study

The preprint servers medRxiv and bioRxiv collate all submitted preprints about COVID-19 research (see https://connect.biorxiv.org/relate/content/181). The population of cases that we studied are the preprints that are published on medRxiv and bioRxiv and classified as COVID-19 research by these servers. Our sampling frame consists of all COVID-19 preprints that were available from the preprint servers between 19 January 2020 and 31 January 2021. We selected 19 January 2020 as the starting date, because this is the date of the oldest COVID-19 preprint on the servers (accessed on 2 February 2021). The sampling frame also contains information about characteristics of the preprints, such as the number of authors, the subject category, the server a preprint was published on, and the date a preprint was published. These four characteristics were used to draw a stratified random sample of 1200 COVID-19 preprints to ensure that the characteristics of the population of preprints are represented as closely as possible in the sample.

The strata used in this sampling procedure were: the preprint server (medRxiv or bioRxiv), subject category as identified by the preprint servers, month of the year a preprint was published, and a categorical variable indicating the number of authors of a preprint (categories 1, 2, 3–10, 11–∞). We included strata for preprint server and subject category as the prevalence of statistical reporting inconsistencies may differ between research areas. Month of the year was included to take potential differences into account between preprints published at the start of the pandemic or more than one year later. The categories for the number of preprint authors were selected to reflect possible differences in the sense of responsibility authors may feel to double-check the reported statistical results. It is imaginable that there is a difference between single-authored and multi-authored preprints, because co-authors may check the statistical results in multi-authored preprints. It is also imaginable that there is a diffusion of responsibility as the number of authors increases, which is reflected by the different categories for 2, 3–10 and 11–∞ authors.

We also have a sampling frame of all non-COVID-19 preprints published between 19 January 2020 and 31 January 2021. The same strata as above were used to match each sampled COVID-19 preprint to a comparable non-COVID-19 preprint. That is, for each COVID-19 preprint we selected a non-COVID-19 preprint that is published on the same preprint server, in the same subject category, in the same month of the year, and with the same category of number of authors. The most recent version of a COVID-19 preprint was always downloaded, and we also included the version number of the COVID-19 preprint for selecting a matching non-COVID-19 preprint. If multiple non-COVID-19 preprints ended up in the selection, we randomly sampled one of these preprints. We only extracted statistics from a matching preprint if the corresponding COVID-19 preprint contained statistical results of which we could check the consistency. In the case that a COVID-19 preprint contained statistical results but the matching preprint did not, we continued randomly sampling a matching preprint until we had sampled one that contained statistical results.

In the case that there were no matching non-COVID-19 preprints for a certain COVID-19 preprint, we relaxed the month in which a preprint was published by also searching for matching preprints in adjacent months. If there were also no non-COVID-19 preprints published with these characteristics in adjacent months, we again searched for preprints published in the same month as the COVID-19 preprint but now relaxing the characteristic reflecting the number of authors by assessing whether a non-COVID-19 preprint is present in adjacent categories. Third, if this did not result in a matching non-COVID-19 preprint, we searched for preprints in the same category reflecting the number of authors in the COVID-19 preprint but published on the other preprint server. Finally, in the rare cases where this did not result in a match, we looked for a non-COVID-19 preprint with the same subject category and version number, but relaxed all other characteristics. To verify that the matching procedure worked as planned, we manually checked whether a non-COVID-19 preprint was indeed a matched case for the first 50 sampled preprints.

**Figure 1.**
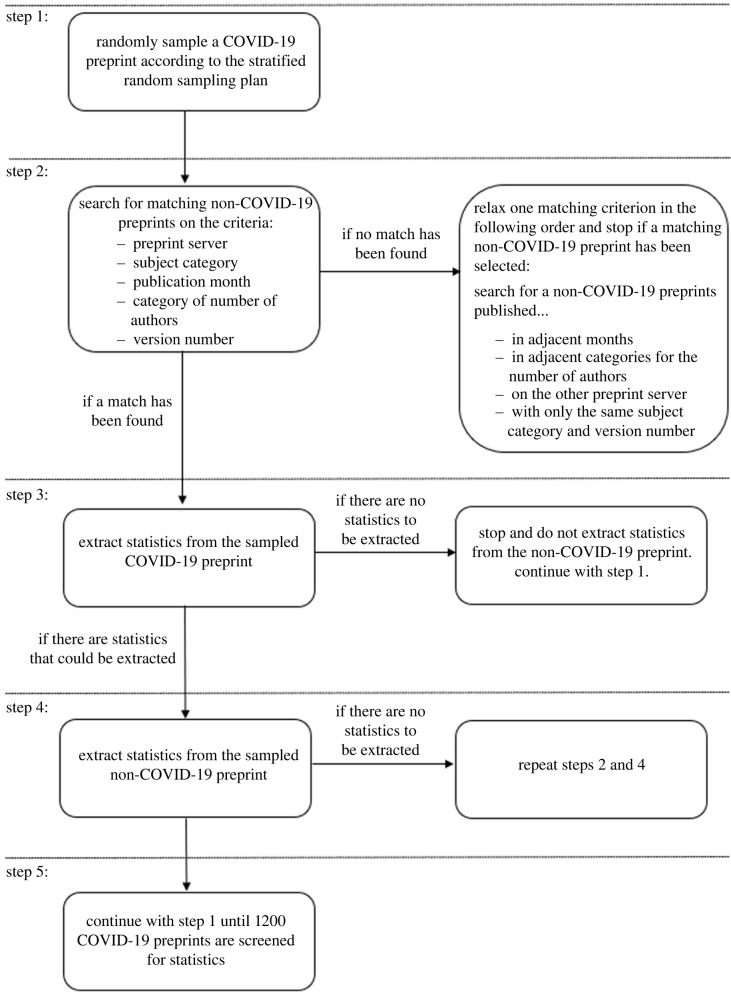
Overview of the stratified sampling and matching procedure.

Figure 1 provides an overview of the stratified sampling and matching procedure. We programmed the stratified sampling procedure and all analyses in the statistical software R (v. 4.0.3; [24]). R code for the stratified sampling procedure and matching procedure is available at https://osf.io/bmkew/ and https://osf.io/6rhu9/.

### 2.2. Data extraction

The dependent variable in our study is the internal consistency of a reported statistic. A statistical reporting inconsistency arises when numbers belonging to a set do not match. For example, when a paper states ‘7% of the patients died in the hospital (5/100)’, it is clear that these numbers are not internally consistent: 5/100 is 5%, not 7%. In our study, we manually extracted^1^ and subsequently assessed the internal consistency of the following types of statistics:

— Reported percentages should match the accompanying fraction (e.g. 5% versus 5/100).
— Reported test sensitivity/specificity/accuracy/positive predictive value/negative predictive value should match reported true positive/true negative/false positive/false negative rates.
— Reported total sample size should match reported subgroup sizes.
— Reported marginal values in frequency tables should match cell values.
— Reported *p*-values should match reported test statistics and degrees of freedom.
— Reported odds ratios/risk ratios/risk differences should match values reported in the associated frequency table.

We extracted reported statistics regardless of whether these came from a primary or secondary analysis. However, we did not extract reported statistics if the preprint contained insufficient information to assess the internal consistency. We also did not extract other types of statistics than the ones mentioned above (e.g. effect sizes such as explained variance in an ANOVA or regression analysis and results of Bayesian analyses), because these types of statistics are usually not reported with enough detail to allow a check for internal consistency. If more than two tables were reported in a preprint, we randomly selected two tables to potentially extract statistics from. We did not extract statistics reported in appendices or electronic supplementary material. See our coding protocol (https://osf.io/n4u5g) for details.

To check and improve inter-rater reliability of the coding procedure, the two research assistants responsible for statistics extraction both coded the same 50 preprints. We assessed inter-rater reliability by verifying that both coders have extracted the same statistics from the preprints. Specifically, we did two things. First, we calculated the correlation between the number of statistics extracted by each coder (*r* = 0.987). Second, we calculated the median difference between the number of extracted statistics of each coder per preprint (median = 2; interquartile range (IQR) = 6). Moreover, a meeting was organized with the two research assistants and one of the principal investigators to evaluate the data extraction procedure for these 50 preprints. Differences in extracted statistics by the two research assistants were discussed in this meeting in order to improve inter-rater reliability even further. In the end, a total of three research assistants extracted statistics. The additional coder was trained by the original two. Given the high inter-rater reliability in the first round of coding, we did not do another round of double-coding preprints to reassess inter-rater reliability.

The extracted data were filled out in a spreadsheet (see https://osf.io/ufcw6 for the template and https://osf.io/a5xmz/ for the filled out spreadsheets), and we ran two R scripts (https://osf.io/er6bg and https://osf.io/8kdzt) to automatically recompute the reported statistic from the other reported information. If the reported and recomputed statistics did not match, the result was marked as inconsistent. We took rounding into account^2^ and only compared the statistics after rounding using the same number of decimals as the reported statistic. If the R script flagged a result as an inconsistency, we manually verified this in the preprint to decrease the probability that we wrongly classified statistics as inconsistencies.

In this operationalization of statistical reporting inconsistencies, we did not take into account the size of the discrepancy, because it is often not possible to determine which of the reported results in the set is/are incorrect. To illustrate, say that a paper reports the following statistic: ‘*t*_28_ = 2.2, *p* = 0.063’. The recalculated *p*-value based on the test statistic and degrees of freedom is 0.036. This could be considered a relatively large discrepancy: the recalculated *p*-value is almost twice as small as the reported *p*-value, and it falls on the other side of the conventional significance level of 0.05. However, without the raw data we have no way of knowing which of the three reported results is incorrect. It could also be the case that the *p*-value is correctly reported, but there is an error in the test statistic and the recalculated test statistic is 1.9 instead of 2.2. This discrepancy is much smaller (percentage-wise) than the discrepancy in the recalculated *p*-values, even though we looked at the same set of inconsistent numbers. Therefore, we decided to only record whether a statistic is consistent or inconsistent, and not try to determine the size of the discrepancy.

### 2.3. Analysis

We tested our hypothesis using a logistic multi-level model, because the dependent variable in our study is dichotomous (whether a statistical result is inconsistent or not). This model also takes into account any dependencies between inconsistencies in results within a single preprint. We indicate a statistical result with *i* and a preprint with *j* such that the statistical model to test our hypothesis is

$$\mathrm{logit}(\pi_{ij})=\gamma_{00}+\gamma_{01}\mathrm{covid}+\mu_{0j},$$

where *π_ij_* is the probability of an inconsistency in statistical result *i* of preprint *j,* $\gamma_{00}$ denotes the intercept, $\gamma_{01}$ is the parameter of interest testing whether COVID-19 preprints contain more or fewer statistical inconsistencies than non-COVID-19 preprints (i.e. covid indicates whether the preprint was about COVID-19 (1) or not (0)), and $\mu_{0j}$ denotes the random effect that follows a normal distribution with mean 0 and variance $\tau^{2}$. Our primary interest is estimating $\gamma_{01}$ and testing whether this parameter is different from zero, which would indicate that results are either more or less likely (instead of equally likely) to be inconsistent if they are reported in a COVID-19 preprint than in a non-COVID-19 preprint. We retain a significance level of *α* = 0.05, because we believe a 0.05 probability of making a Type-I error is acceptable in this study. Furthermore, a significance level of *α* = 0.05 in combination with our sample size enables us to detect an odds ratio of approximately 1.38 with 80% power (see electronic supplementary material, 1 at https://osf.io/7kcja), which we consider practically relevant.

Next to conducting a frequentist hypothesis test, we also employed Bayesian hypothesis testing. We computed approximated adjusted fractional Bayes factors [27] to compare two models with each other where *γ*_01_ = 0 and *γ*_01_ ≠ 0 using the default implementation in the R package BFpack ([28]; v. 1.0.0). Comparing these two models is the Bayesian equivalent of the two-tailed frequentist hypothesis test that we propose. The approximated adjusted fractional Bayes factor uses a minimal fraction of the available data to train a non-informative normally distributed prior and approximate the marginal likelihood of the tested hypotheses.

We also ran another logistic multi-level model where we extended the model above by including control variables to study whether the estimated effect is affected by including other variables in the model. Note that the parameter estimated in this second model is different from the parameter in the model without control variables, because the parameter in the second model refers to the effect controlled for the other variables. All control variables were measured at the preprint level. The control variables that were included are: the number of authors of a preprint (continuous variable), the number of days a preprint was published after the first COVID-19 preprint was published (19 January 2020), and the total number of extracted statistics in a preprint. The number of authors of a preprint and number of days a preprint was published after 19 January 2020 were included for the same reasons as using these variables for creating strata in the stratified random sampling procedure. The total number of extracted statistics in a preprint was included to take into account that a statistical inconsistency is more likely to occur if many statistics are reported in a preprint. We again tested whether there is a difference in how likely a statistical reporting inconsistency is in a COVID-19 preprint compared with a non-COVID-19 preprint using the outlined frequentist hypothesis test above (with *α* = 0.05) as well as by computing the proposed Bayes factor. We used the R package lme4 ([29]; v. 1.1-33) for fitting the logistic multi-level models. R code of our planned analyses is available at https://osf.io/9emzb. We had to make small changes to the R code to ensure that the preregistered code could be applied. This modified R code is available at https://osf.io/r3892.

## 3. Results

### 3.1. Descriptives

Table 2 describes our final sample. Of the 984 sampled COVID-19 preprints and 784 screened non-COVID-19 preprints, we could extract statistics in 533 preprint pairs (54.2% for COVID-19 and 68.0% for non-COVID-19). We initially planned to sample 1200 COVID-19 preprints, but it turned out that more preprints than the expected 45% specified in the power analysis contained statistical results. Hence, we stopped data collection when 1200 × 0.45 = 540 COVID-19 preprints with statistical results were coded. We ended up with 533 preprints, because some preprints were accidentally coded twice and after manually verifying the extracted statistics in the case of an inconsistency we found that some statistics should not have been included in the sample. The vast majority of preprints came from medRxiv for COVID-19 (462, 86.7%) and non-COVID-19 (482, 90.4%) preprints. The mean number of statistics in a preprint was slightly higher in COVID-19 preprints than non-COVID-19 preprints (42.2 versus 34.0, respectively), but the medians and IQRs were similar (table 2).

**Table 2.** Descriptive statistics of the final sample of preprints under analysis.

	COVID-19	non-COVID-19
preprints with statistics	533	533
number of statistics in preprints with statistics	mean: 42.2	mean: 34.0
	median: 13 (IQR = 50)	median: 12 (IQR = 41)
authors on a preprint	mean: 11.1	mean: 10.3
	median: 8 (IQR = 10)	median: 8 (IQR = 8)
preprints from medRxiv and bioRxiv (%)	medRxiv: 462 (86.7%)	medRxiv: 482 (90.4%)
	bioRxiv: 71 (13.3%)	bioRxiv: 51 (9.6%)

The matching procedure yielded 1039 matched preprints that met all matching criteria (i.e. published on the same preprint server, in the same subject category, in the same month of the year, and with the same category of number of authors). In 115 cases, a matching preprint could not be found in which all criteria were met, but there was a matching preprint available in the adjacent month to the month the COVID-19 preprint was submitted. In 36 cases, a matching preprint was found where only the criterion of the number of authors needed to be relaxed such that a matching preprint was selected that belonged to an adjacent category. In 55 cases, the matching was only done on version and subject category.^3^

We realized during the data analysis that a bug in our code caused the correct matching preprint to be coded in only 489 (91.7%) preprints. In the remaining 44 (8.3%) preprints, a non-COVID-19 preprint was coded that was not a match for the COVID-19 preprint. Since the non-matching preprints still came from the relevant preprint servers and general timeframe, we decided to report the results based on all coded preprints in the paper. This way, we still make use of all available evidence. Additionally, a sensitivity analysis based on the 489 correctly matched preprints showed that it did not change the conclusions of our study (see electronic supplementary material, tables S6 and S7).

**Figure 2.**
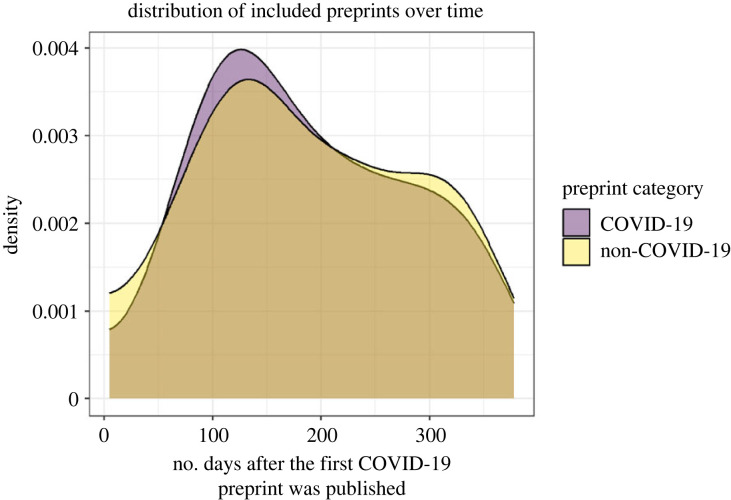
A Gaussian kernel density plot of included preprints over time per preprint category (COVID-19 versus non-COVID-19 preprint).

We compared the preprints in the COVID-19 and non-COVID-19 group on the matching characteristics and confirmed that the mean number of authors on a preprint was close for COVID-19 and non-COVID-19 preprints (11.1 versus 10.3, respectively, with identical medians of 8). We also obtained a similar distribution of preprints over time for the COVID-19 and non-COVID-19 preprints (figure 2). Finally, the distribution of the COVID-19 and non-COVID-19 preprints over the subject categories was similar to well. We report details on the included preprints per subject category in electronic supplementary material, table S1.

In total, across COVID-19 and non-COVID-19 preprints we initially extracted 40 838 statistics. Applying our R script to check the statistics for inconsistencies initially yielded 4396 inconsistencies (10.8%). We then manually verified all inconsistencies. In 205 cases, the statistics should not have been extracted (4.7%; e.g. percentages in tables that were coded as marginals), and in 441 cases (10.5% of the 4191 remaining flagged inconsistencies) the inconsistency was due to a mistake in the data extraction. This left us with a total of 40 633 correctly extracted statistics, of which 3750 were actual inconsistencies (9.2%).

**Figure 3.**
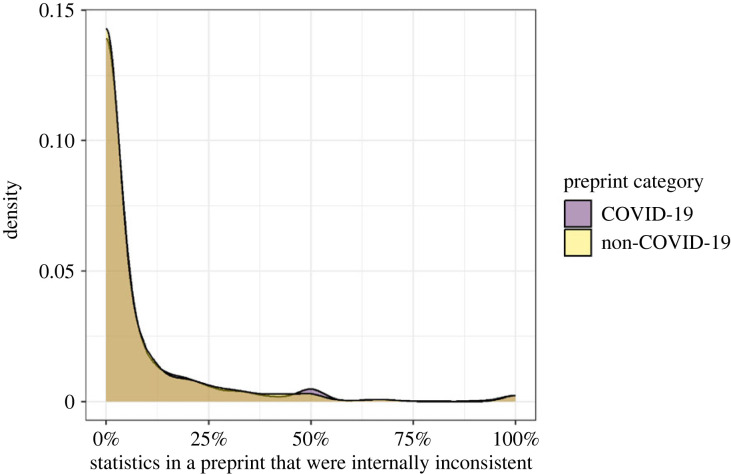
A Gaussian kernel density plot of the percentage statistics within a preprint that was inconsistent per preprint category (COVID-19 versus non-COVID-19 preprint).

Table 3 shows descriptive results on the prevalence of statistical inconsistencies in COVID-19 and non-COVID-19 preprints. In total, 286 of the 533 COVID-19 preprints contained at least one inconsistency, compared with 275 of the 533 non-COVID-19 preprints (53.7% versus 51.6%, respectively). Of the 22 498 statistics extracted from COVID-19 preprints, 2040 (9.07%) were inconsistent, compared with 1710 (9.43%) of the 18 135 statistics from non-COVID-19 preprints. Note that these percentages do not take into account the nested structure of the data: a statistic is likely to be more similar to a statistic in the same preprint than to a statistic in another preprint. We therefore also calculated the percentage of inconsistencies within each preprint separately and then calculated the mean across these percentages. To illustrate: say that in paper A 1 out of the 10 reported statistics was inconsistent (10%) and in paper B 2 out of the 4 statistics were inconsistent (50%). In this example, the mean percentage of inconsistencies in a paper would then be (10 + 50)/2 = 30%. In our data, this mean percentage of inconsistencies in COVID-19 preprints was 9.8%, compared with 9.4% in non-COVID-19 preprints. Additionally, figure 3 shows considerable overlap in the COVID-19 and non-COVID-19 distributions of the percentage of inconsistencies within preprints. Taken together, these descriptives indicate that the difference in inconsistencies between COVID-19 and non-COVID-19 preprints is small in the current sample.

**Table 3.** Prevalence of inconsistent statistics in COVID-19 and non-COVID-19 preprints.

	COVID-19	non-COVID-19	
total number of preprints	533	533	
number of preprints with at least one inconsistency	286 (53.7%)	275 (51.6%)	difference in %: 2.1
number of statistics	22 498	18 135	
overall number of inconsistencies (%)	2040 (9.1%)	1710 (9.4%)	difference in %: −0.3
mean % inconsistencies in a preprint (takes nesting into account)	9.8%	9.4%	difference in %: 0.4

### 3.2. Confirmatory analyses

The results of our frequentist confirmatory analyses can be found in table 4. In the model without control variables, we found no significant difference in the probability that a statistic was inconsistent between COVID-19 and non-COVID-19 preprints: *γ*_01_ = 0.016, 95% CI [−0.216, 0.248], *Z* = 0.132, *p* = 0.895. That is, the odds for an inconsistency in a COVID-19 preprint are exp(0.016) = 1.016 times the odds of an inconsistency in a non-COVID-19 preprint. The 95% CI of this odds ratio is [0.806; 1.281] suggesting that there is no evidence for meaningful differences in inconsistency rates between COVID-19 and non-COVID-19 preprints. The Bayesian hypothesis test was in line with this result and indicated strong evidence [30] that the prevalence of inconsistencies is the same in COVID-19 and non-COVID-19 preprints. Specifically, given these data, the model where statistical inconsistencies are equally prevalent in COVID-19 and non-COVID-19 preprints is 51.4 times more likely than the unconstrained model where the prevalence differs between COVID-19 and non-COVID-19 preprints (BF_01_ = 51.4). The posterior probability that *γ*_01_ equals zero was 0.963.

**Table 4.** Results of estimating the multi-level logistic regression models that predict whether a statistical result is inconsistent or not based on whether a preprint is on COVID-19 or not, with random intercepts for preprints. Models 1 and 2 are without and with the control variables, respectively.

			model 1					model 2				
effect	group	term	est.	s.e.	Z	*p*	95% CI	est.	s.e.	Z	*p*	95% CI
fixed		intercept	−2.943	0.088	−33.304	<0.001	[−3.117, −2.770]	−2.913	0.164	−17.769	< 0.001	[−3.233, −2.592]
		COVID-19	0.016	0.118	0.132	0.895	[−0.216, 0.248]	0.038	0.118	0.324	0.746	[−0.193, 0.269]
		no. of authors						−0.004	0.006	−0.671	0.502	[−0.016, 0.008]
		no. of days after publication of first preprint						0.000	0.001	0.638	0.524	[−0.001, 0.002]
		no. of extracted statistics						−0.001	0.001	−1.463	0.143	[−0.003, 0.000]
random	preprint	s.d. of the intercept	1.422					1.405				

*Note.* est. = estimate. s.e. = standard error. CI = Wald-based confidence interval. s.d. = standard deviation. no. = number.

When controlling for the number of authors on a preprint, the number of days after publication of the first COVID-19 preprint, and the number of statistics in a preprint, we also found no difference between the prevalence of reporting inconsistencies between COVID-19 and non-COVID-19 preprints: *γ*_01_ = 0.038, 95% CI [−0.193, 0.269], *Z* = 0.324, *p* = 0.746 (table 4).^4^ The conclusions based on the results of the Bayesian hypothesis test also did not change when including the control variables (BF_01_ = 49.2). The posterior probability that *γ*_01_ equals zero was 0.961.

Adding the control variables to the model resulted in estimation problems in the frequentist analysis because the range of the scores on the control variables was large (e.g. the number of statistics in a preprint ranged from 1 to 801). This could have resulted in incorrect estimates and statistical inferences for the preregistered model. Therefore, as an additional, unregistered sensitivity analysis we re-estimated the model but this time after standardizing the control variables. The results of fitting the preregistered and non-preregistered models yielded comparable results suggesting that the result of the preregistered model is robust. Hence, we report the results of the preregistered model in table 4 and report the results of the non-preregistered model in the electronic supplementary material (https://osf.io/7kcja, table S2). In the frequentist analysis, we found no evidence for a difference in the probability that a statistic was inconsistent between COVID-19 and non-COVID-19 preprints. We also redid the Bayesian analysis of the model with the standardized control variables, and the results were again comparable to the preregistered analyses (see electronic supplementary material, table S5). Again, we found strong evidence in favour of the null model of no difference.

### 3.3. Exploratory: inconsistencies per type of statistic

**Figure 4.**
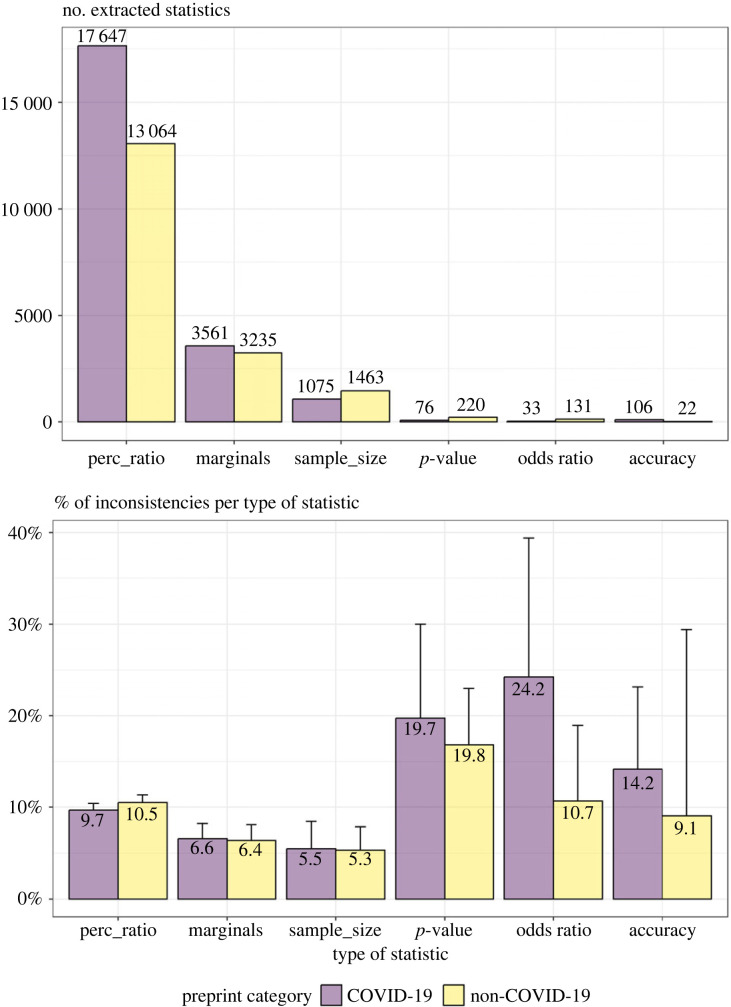
The top panel shows the number of extracted statistics per type of statistic per preprint category (COVID-19 versus non-COVID-19 preprint). The results based on COVID-19 preprints are indicated by purple bars and those of non-COVID-19 preprints by yellow bars. The bottom panel shows the percentage of extracted statistics that was internally inconsistent per preprint category. Error bars indicate standard errors of the percentages.

We checked the consistency of six different types of statistics. It is possible that some are inherently more error-prone than others, for instance because the associated statistical output is more complex or because they consist of a larger set of numbers that all have to be copied correctly to the manuscript. If one type of statistic occurs more in COVID-19 preprints than in non-COVID-19 research, or vice versa, it may confound our results. To explore this question, we calculated the number of extracted statistics and percentage of inconsistencies per type of statistic, split up for COVID-19 and non-COVID-19 research (figure 4).

Overall, we found the highest prevalence of inconsistencies in the sample for *p*-values (test statistics and degrees of freedom not matching the *p*-value), odds ratios (cell frequencies not matching the odds ratios), and accuracy statistics (true/false positives/negatives not matching the sensitivity, specificity, positive or negative predicted value), as compared with lower inconsistency rates in percentages (ratios not matching percentages), marginals (cell values not matching marginal values in frequency tables), and sample sizes (subgroup sizes not matching total sample size). We speculate that this could reflect a relation between the complexity of the statistic and error-proneness (in line with the findings of [21]). Inconsistency rates for all types of statistics except for percentages/ratios were slightly higher for COVID-19 than non-COVID-19 research.

We note, however, that the prevalence of the different types of statistics differed greatly. The higher inconsistency rates in *p*-values, odds ratios and accuracy statistics represent a small number of cases, which makes it difficult to extrapolate these percentages. To illustrate, we found that 24.2% of the odds ratios in COVID-19 research were inconsistent. This seemingly staggering percentage is put into perspective if we consider that it effectively came down to eight inconsistencies in total.

## 4. Discussion

In this preregistered natural experiment, we compared the prevalence of statistical reporting inconsistencies in COVID-19 preprints and in matched non-COVID-19 preprints. We found strong evidence for no difference in the prevalence of statistical reporting inconsistencies between these two groups.

Our hypothesis was two-sided: on the one hand, we speculated that inconsistencies could be lower in COVID-19 research, because the perceived importance of the research may have caused researchers to display more scrutiny in writing up their results. Conversely, we also considered it possible that the prevalence of inconsistencies could be higher in COVID-19 research, because time pressure and societal pressure could have led to hasty and sloppy research and reporting. The latter would be in line with the previously found pattern suggesting that COVID-19 research was less rigorous and contained more methodological problems [12–16]. Our results did not support either notion: the high pressure and high speed with which COVID-19 research was conducted did not seem to be associated with the quality of statistical reporting.

To our knowledge, our study was the first to assess statistical reporting inconsistencies in this context, and therefore adds to the broader body of literature studying the potential effects of high-speed science. Even though our design was observational and not experimental, our results may help shed some light on the specific stages of research that are affected if researchers are put under pressure and/or try to conduct and publish studies as fast as possible. It may be the case that ‘high-speed publishing’ only affects methodological quality but not statistical reporting. Generally speaking, designing a study and collecting data are more time-consuming than writing up an article, so it may be the case that authors mainly tried to speed up those first research phases (to their detriment), and did not change the way they reported their findings.

### 4.1. Limitations

Our design came with certain limitations. First, we specifically focused on the internal consistency of reported statistics. This means that we could only check results for which all relevant information was reported. However, it is imaginable that when the statistical analyses are done less carefully, the reporting may also be done less carefully and not all information might be there to allow a consistency check. Relatedly, with our procedure, we were not able to spot mistakes or issues in the way the raw data were collected, processed, or analysed.

Second, our population included preprints published on two specific preprint servers: bioRxiv and medRxiv. We chose these servers because they provided a curated list of COVID-19 research. This allowed us to draw a probability sample and search for matching non-COVID-19 preprints. It is possible that preprints published on other preprints servers differ from the included preprints in systematic ways. For example, most preprints on bioRxiv and medRxiv are from biology and the health sciences, whereas COVID-19 research also took place in other scientific fields (e.g. psychology), and are therefore uploaded to other preprint servers (e.g. PsyArXiv). However, since bioRxiv and medRxiv (the latter, specifically) have played the biggest role in distributing COVID-19 preprints [2], we think they were the most relevant servers to study.

Third, we do not know to what extent our findings generalize to published papers. An important difference between preprints and published papers is that the latter are peer reviewed. One could expect that the overall inconsistency rates in published papers would therefore be lower than in unreviewed preprints (e.g. Carneiro *et al.* [31], who found a small improvement in reporting quality in peer-reviewed papers compared with the corresponding preprints). However, given the high prevalence of statistical reporting inconsistencies in the published literature [21,22], it seems that the peer review process has not been sufficiently effective in correcting statistical reporting errors. It may be possible to compare the inconsistency rates in preprints we found in this study to the inconsistency rates in published papers found by Georgescu & Wren [21]. However, this cannot be directly compared since our approaches are not fully equivalent: Georgescu and Wren looked only at statistical ratios (hazard ratio, odds ratio, and relative risk) in abstracts, whereas we included a wider range of statistics reported in the full text. One avenue for future research is to use our materials to study (the same types of) statistical inconsistencies in published studies. This would allow for a more direct comparison with our results. Additionally, future research could check whether the statistical inconsistencies that we observed in preprints are still there in the published versions of these preprints. If the inconsistencies are no longer in the published versions, this would increase trust in peer-review, since it is quite likely that the inconsistencies were spotted and corrected during the review process.

Fourth, we selected our matched control group in order to compare the COVID-19 preprints with ‘research as usual’. However, the included non-COVID-19 preprints were uploaded during the same time period as the COVID-19 preprints, and probably contained many studies that were conducted during the pandemic. It is possible that the authors of the selected preprints (both COVID-19 and non-COVID-19) experienced challenges in doing their research (e.g. working from home, having limited access to laboratories, and having difficulties finding and/or working with participants due to social distancing and related measures), which could have led to higher work pressure and higher inconsistency rates in *both* groups. However, we are unable to determine this based on our data. That being said, we do note that the inconsistency rates in COVID-19 and non-COVID-19 preprints are similar to the inconsistency rates found in psychology research over the years (approx. 10% of statistics is inconsistent; [22]), so we have no immediate reason to suspect that reporting inconsistencies increased during the pandemic.

### 4.2. Implications for understanding statistical reporting inconsistencies

This work has theoretical implications for understanding how statistical inconsistencies arise. Statistical inconsistencies appear to be widespread across different scientific fields [19–23] but little is known about what the main reasons are that such inconsistencies arise. Understanding where and why the problem of statistical misreporting occurs is important to design interventions to prevent statistical errors in future studies. Are inconsistencies a result of insufficient statistical knowledge and should we invest more in statistical education? Are inconsistencies conscious or unconscious misrepresentations of the data to draw more favourable conclusions and should we prioritize integrity training? Are inconsistencies simply random typos that could be solved by steering authors towards software such as RMarkdown?^5^

In this study, we hypothesized that time pressure and strong societal interests could affect reporting quality. As a proxy for these factors, we compared COVID-19 and non-COVID-19 preprints, because these types of research arguably differed in these aspects. However, we found strong evidence that there is no difference in inconsistencies between COVID-19 and non-COVID-19 preprints, which provides preliminary, indirect evidence against the notion that time pressure and/or societal pressure may lead to sloppy statistics reporting. So far, previous literature provides some evidence that inconsistencies are associated with a lack of data sharing ([32]; but see [33]), fewer authors on a paper ([21]; but see [34]), lower journal impact factor [21], and more complex statistics ([21]; also in line with our exploratory findings in this paper). There are indications that there is a systematic tendency of inconsistencies to be in favour of the authors' conclusions [19,35], but the bulk of reporting inconsistencies does not seem to have a direct effect on statistical conclusions [22].

While these studies have suggested several potential avenues for providing interventions aimed at preventing statistical inconsistencies, not many interventions have been designed, tested and implemented. One study that did assess a potential intervention looked at *statcheck* as a possible tool for preventing statistical inconsistencies [36]. *statcheck* is a free R package and web app that automatically extracts *p*-values from articles and checks whether they match their accompanying test statistic and degrees of freedom [37]. The authors found fewer inconsistencies in articles published in journals that used *statcheck* in their peer review process compared with matched control journals. This indicates that automated screening tools during the peer review process could potentially help decrease statistical reporting inconsistencies. Because *statcheck* is currently only able to process APA-formatted manuscripts, it would be valuable to extend *statcheck* so that it can also automatically check the statistics in papers with different formats, like the ones we assessed in this paper. We note that the lack of general reporting guidelines with respect to statistical results severely hampers automated screening. Our current dataset of over 40 000 manually extracted statistics may serve as a validation set for future automated screening tools or as training data for methods based on artificial intelligence.

We note that—to our knowledge—all research on statistical reporting inconsistencies has been observational (descriptive or correlational). It would be useful to extend the current literature by performing randomized controlled trials that systematically investigate different potential causes of inconsistencies and/or directly test interventions.

### 4.3. Implications for the medical and biological sciences

Our findings also have practical implications for the medical and biological sciences. We note that the overall inconsistency rate we found in our sample of medical and biological preprints was substantial: roughly 1 in 11 reported statistics was internally inconsistent. Since we did not assess the size of the discrepancies or whether an inconsistency pertained to a key result or not, we do not know to what extent the discovered inconsistencies substantially affected conclusions. However, any inconsistency in a statistical result renders it ‘irreproducible’ [38,39], which means that the reported result cannot be traced back to the underlying data. Reproducibility is a basic, necessary requirement for robust science, because it allows other researchers to verify results: if we do not know where a number came from, how can we interpret it?

Although much is still unclear about the specific causes of statistical inconsistencies in particular, we can make some recommendations to improve general analytical reproducibility. Low hanging fruit would be for authors themselves to double-check the internal consistency of their reported statistics, where possible with automated tools such as *statcheck* [37]. However, to really improve reproducibility, authors should share (anonymized) raw data and analysis code, preferably in line with the FAIR principles (findable, accessible, interoperable and reusable; [40]). Making data and code available does not only allow others to rerun the analyses and spot potential mistakes, it also allows for sensitivity analyses and in some cases might even allow for answering or generating new research questions.

## 5. Conclusion

The COVID-19 pandemic created a unique setting to study the effect of high-speed scientific publishing on the statistical reporting quality. In this study, we found no indication that such high-speed science is related to the number of statistical reporting inconsistencies. Taken together with previous research, our results suggest that COVID-19 research may on average have more methodological problems than non-COVID-19 research, but there is no difference in the statistical reporting quality. That said, the overall prevalence of statistical reporting inconsistencies in the biological and health sciences shows that the scientific community still has room for improving the reproducibility of published research.

## Data Availability

All materials of this study (including data and R code of the analyses) are available on the OSF page of this project: https://osf.io/tdfgq/.

## Footnotes

1 Note the tool *statcheck* [25] that automatically extracts null hypothesis significance tests and checks the consistency of reported *p*-values cannot be used here, because it can only detect statistics reported in APA style [26], which is not used in the majority of preprints submitted to bioRxiv and medRxiv.

2 Different software packages sometimes maintain different rounding rules. Specifically, depending on which software is used for rounding (or if done manually), a value ending in 5 can be either rounded up or down. To take this into account, we will round recalculated values ending in 5 in both directions and if either matches the reported value consider it correct.

3 Note that the total number of matched preprints is equal to 1039+115+36+55=1245. This differs from the total number of sampled preprints (1231), because some preprints were selected twice as matching preprint. These preprints are only included once in the data, and we made sure that matching preprints could only be selected once when we became aware of this issue.

4 We included the number of preprint authors as a control variable, because statistics can be checked by co-authors in a multi-authored preprint. However, a large number of co-authors could also lead to a diffusion of responsibility such that no author checks the reported statistics. This is in line with a quadratic effect of the number of preprint authors, but we preregistered a non-squared effect of number of preprint authors. Hence, we conducted a sensitivity analysis where we also included the quadratic effect of the number of preprint authors in the model. The results of the analyses did not change our conclusions. See Tables S3 and S4 in the electronic supplementary material (https://osf.io/7kcja) for these results.

5 RMarkdown allows to conduct the analyses inside the paper such that the results of the analyses can automatically be inserted in the text rather than copy-and-pasted from the output.
